# Determining the prognosis of Lung cancer from mutated genes using a deep learning survival model: a large multi-center study

**DOI:** 10.1186/s12935-023-03118-y

**Published:** 2023-11-04

**Authors:** Jie Peng, Lushan Xiao, Hongbo Zhu, Lijie Han, Honglian Ma

**Affiliations:** 1 Department of Medical Oncology, The Second Affiliated Hospital, Guizhou Medical University, Kaili, China; 2 Hepatology Unit, Department of Infectious Diseases, Nanfang Hospital, Southern Medical University, Guangzhou, China; 3 Department of Medical Oncology, The First Affiliated Hospital, Hengyang Medical School, University of South China, Hengyang, China; 4 Department of Hematology, The First Affiliated Hospital of Zhengzhou University, Zhengzhou, China; 5 Department of Radiation Oncology, Cancer Hospital of the University of Chinese Academy of Sciences, Hangzhou, China

**Keywords:** Deep learning, Lung cancer, Mutated gene, Prognosis, Immunotherapy

## Abstract

**Background:**

Gene status has become the focus of prognosis prediction. Furthermore, deep learning has frequently been implemented in medical imaging to diagnose, prognosticate, and evaluate treatment responses in patients with cancer. However, few deep learning survival (DLS) models based on mutational genes that are directly associated with patient prognosis in terms of progression-free survival (PFS) or overall survival (OS) have been reported. Additionally, DLS models have not been applied to determine IO-related prognosis based on mutational genes. Herein, we developed a deep learning method to predict the prognosis of patients with lung cancer treated with or without immunotherapy (IO).

**Methods:**

Samples from 6542 patients from different centers were subjected to genome sequencing. A DLS model based on multi-panels of somatic mutations was trained and validated to predict OS in patients treated without IO and PFS in patients treated with IO.

**Results:**

In patients treated without IO, the DLS model (low vs. high DLS) was trained using the training MSK-MET cohort (HR = 0.241 [0.213–0.273], *P* < 0.001) and tested in the inter-validation MSK-MET cohort (HR = 0.175 [0.148–0.206], *P* < 0.001). The DLS model was then validated with the OncoSG, MSK-CSC, and TCGA-LUAD cohorts (HR = 0.420 [0.272–0.649], *P* < 0.001; HR = 0.550 [0.424–0.714], *P* < 0.001; HR = 0.215 [0.159–0.291], *P* < 0.001, respectively). Subsequently, it was fine-tuned and retrained in patients treated with IO. The DLS model (low vs. high DLS) could predict PFS and OS in the MIND, MSKCC, and POPLAR/OAK cohorts (*P* < 0.001, respectively). Compared with tumor-node-metastasis staging, the COX model, tumor mutational burden, and programmed death-ligand 1 expression, the DLS model had the highest C-index in patients treated with or without IO.

**Conclusions:**

The DLS model based on mutational genes can robustly predict the prognosis of patients with lung cancer treated with or without IO.

**Supplementary Information:**

The online version contains supplementary material available at 10.1186/s12935-023-03118-y.

## Background

To optimize treatment regimens, predicting the prognosis of patients with lung cancer is vital. Accordingly, gene status has gradually become the focus of prognosis prediction. Based on high-throughput sequencing, multi-panels have been routinely evaluated in clinical treatment, revealing various candidate genes. For instance, the *KRAS-G12C* mutation is associated with poorer outcomes in surgically resected lung adenocarcinoma than wild-type *KRAS* [1]. Meanwhile, the *SMARCA4* mutation is an independent predictive factor for poor prognosis in lung cancers, however, is also associated with immunotherapy (IO) sensitivity [2]. Additionally, mutations in *EGFR*, *STK11*, and *B2M*, or *MDM2* amplification, are related to IO resistance or hyperprogressive disease [3–5], while *TP53*, *KRAS*, and *POLE* mutations are positively associated with a good response in advanced non-small cell lung cancer (NSCLC) [6–9].

Deep learning has frequently been implemented in medical imaging (including magnetic resonance imaging, computed tomography, and positron emission tomography) to diagnose, prognosticate, and evaluate treatment responses in patients with cancer [10–12]. Previous studies have used several genes or immune cell subtypes to develop models to predict IO or chemo-IO responses by machine learning. These studies achieved highly reliable and accurate results [13–15]. However, few deep learning survival (DLS) models based on mutational genes that are directly associated with patient prognosis in terms of progression-free survival (PFS) or overall survival (OS) have been reported, and their potential value remains unclear. Additionally, DLS models have not been applied to determine IO-related prognosis based on mutational genes.

The current study employed a DLS algorithm utilizing a panel of mutated genes to create a robust survival model to identify individuals with lung cancer and good prognosis in several large centers. Based on whole-genome sequencing (WGS), next-generation sequencing (NGS), and whole-exome sequencing (WES) databases, the DLS model was used to predict OS in patients with lung cancer who were treated without IO and to predict PFS in patients with lung cancer who were treated with IO. The predictive ability of the DLS model was compared with that of clinical tumor-node-metastasis (TNM) staging and the COX model. In addition, the ability of the DLS model to predict PFS in those who received IO was compared with that of the COX model, tumor mutational burden (TMB), and programmed death-ligand 1 (PD-L1) expression. A robust survival prediction model based on genomics panels will aid oncologists in implementing appropriate treatment strategies for patients with lung cancer.

## Methods

### Patients treated without IO

#### MSK-MET cohort

A total of 25,775 patients with metastatic cancers were included in the MSK-MET cohort [16]. However, 21,711 with other tumors were excluded, resulting in a final cohort comprising 4064 patients with lung cancer. Additionally, 271 patients had incomplete clinical or survival data and were thus excluded from this study. Ultimately, the data for 3793 patients with lung cancer were analyzed. The MSK-MET cohort was classified into training (*n* = 2504) and inter-validation (*n* = 1289) cohorts; all tumor samples were evaluated by NGS.

#### OncoSG cohort

The OncoSG cohort comprised 305 patients from East Asia countries. Eight patients lacking clinical or survival data were excluded [17]. Hence, 297 patients with lung adenocarcinoma were included in an independent validation cohort. All tumor samples were evaluated by WES.

#### MSK-CSC cohort

This cohort comprised 10,945 patients, of which, 9588 patients with other tumors were excluded [18]. Further, 417 patients without clinical or survival data were excluded. Thus, 940 patients with lung cancer comprised an independent validation cohort. All tumor samples were assessed by NGS.

#### TCGA-LUAD cohort

Among the 566 patients with lung adenocarcinoma, 52 were excluded due to a lack of clinical data (https://www.cell.com/pb-assets/consortium/pancanceratlas/pancani3/index.html). Moreover, 26 patients without complete survival data were excluded. Thus, 488 patients with lung adenocarcinoma comprised an independent validation cohort; all tumor samples were assessed by WGS.

### Patients treated with IO

#### MIND cohort

A total of 247 patients with lung cancer from the Memorial Sloan Kettering Cancer Center (MSKCC) cohort were recruited [19]. All patients received anti-PD-1/PD-L1 treatment. One patient was excluded due to a lack of clinical data. Hence, 246 patients were included in this training cohort. All tumor samples were evaluated by NGS.

#### MSKCC cohort

A total of 349 patients from a clinical trial and retrospective analysis (NCT01454102, NCT01295827) who received anti-PD-1/PD-L1 monotherapy or combinatorial treatment with anti-CTLA4 were included [20]. These patients constituted another validation cohort. All tumor samples were analyzed by NGS.

#### POPLAR/OAK cohort

The POPLAR and OAK studies (NCT01903993, NCT02008227) recruited 1137 patients with advanced or metastatic NSCLC [21, 22]. Patients treated with docetaxel (*n* = 568) and those without blood TMB data (*n* = 140) were excluded. Ultimately, the POPLAR/OAK cohort comprised 429 patients as a validation cohort. All blood samples were tested by NGS.

This study (2023-LUNSHEN-02) was approved by the institutional review board of the Second Affiliated Hospital of Guizhou Medical University and was performed in accordance with the Declaration of Helsinki. Informed consent was obtained from all patients for tissue or blood use.

### Study design

**Fig. 1:**
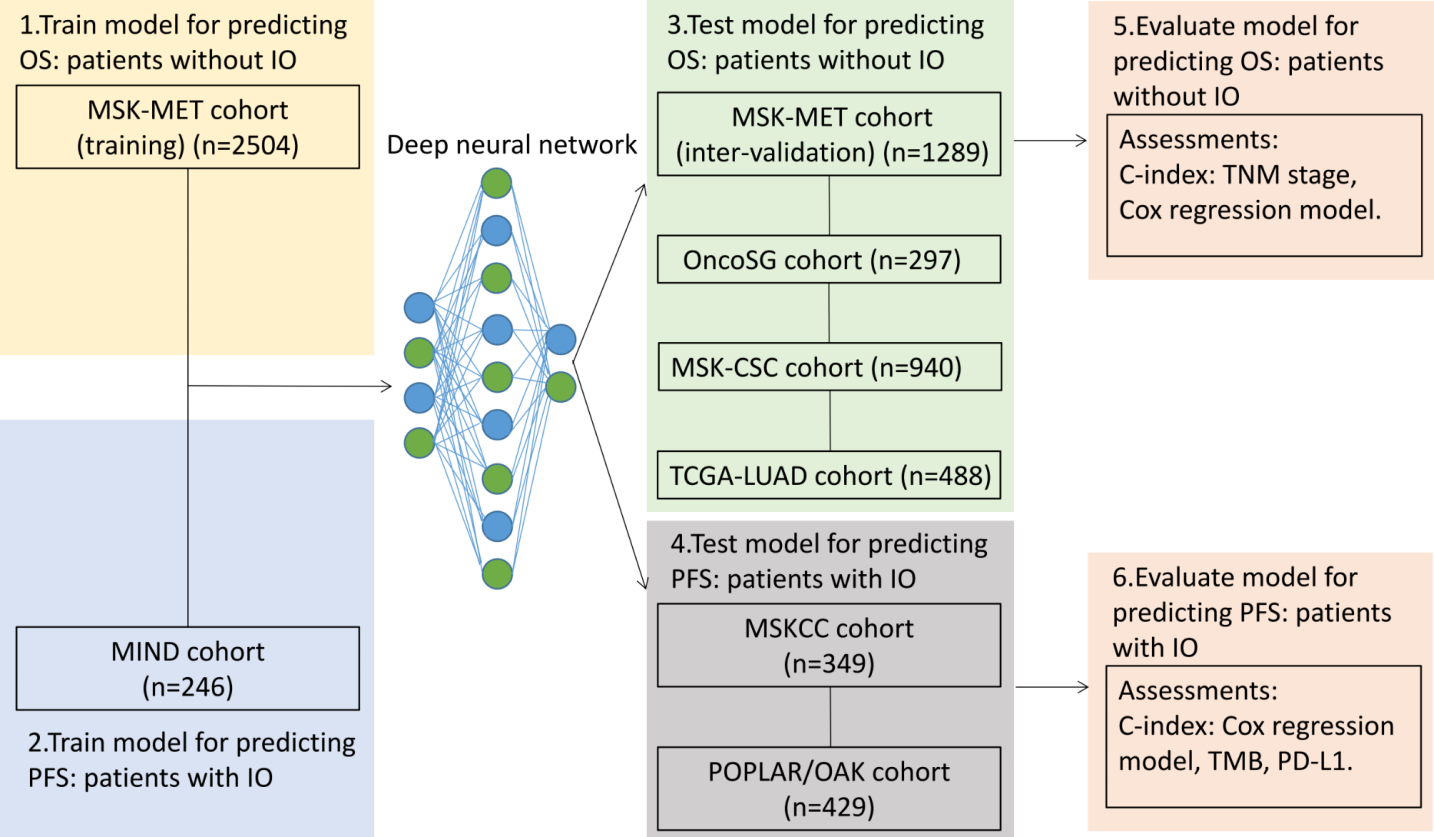
Flowchart of the proposed deep learning survival (DLS) model to determine disease prognosis. The somatic mutational databases were derived from non-small cell lung cancer (NSCLC) samples. In the MSK-MET cohort (training), the selected genes were trained to predict overall survival (OS) using deep learning. After adjusting the training parameters, the DLS models were validated for OS in the MSK-MET (inter-validation), OncoSG, MSK-CSC, and TCGA-LUAD cohorts. The trained DLS model was fine-tuned and re-trained using the MIND cohort. The DLS model was validated in the MSKCC and POPLAR/OAK cohorts. The COX models were analyzed in all patients. The C-indices of the DLS model, COX model, and tumor-node-metastasis (TNM) staging were compared in patients treated without immunotherapy (IO) regarding OS. The C-indices of the DLS model, COX model, tumor mutational burden (TMB), and programmed death-ligand 1 (PD-L1) expression were also compared among patients treated with IO regarding progression-free survival (PFS).

Figure 1 illustrates the flowchart of proposed DLS models for predicting OS and PFS. In the MSK-MET (training) cohort, optimal mutated genes were identified by the least absolute shrinkage and selection operator (LASSO) algorithm based on five-fold cross-validation. The selected genes served as input for training the DLS models to predict OS. The training parameters were adjusted, and the DLS models were validated for OS in the MSK-MET (inter-validation), OncoSG, MSK-CSC, and TCGA-LUAD cohorts. The LASSO algorithm for predicting PFS was also used to select the mutated genes in the MIND cohort in patients treated with IO. The trained DLS model was fine-tuned and retrained in the MIND cohort and, subsequently, tested in the MSKCC and POPLAR/OAK cohorts. The COX models were analyzed in patients treated with and without IO. The performance of the DLS model, COX model, and TNM staging for predicting OS in patients treated without IO was compared (via the C-index). Furthermore, the performance of the DLS model, COX model, TMB, and PD-L1 expression level for predicting PFS was compared among patients treated with IO using the C-index.

### TMB, PD-L1 expression analysis, and selection of optimal mutated genes

**Fig. 2:**
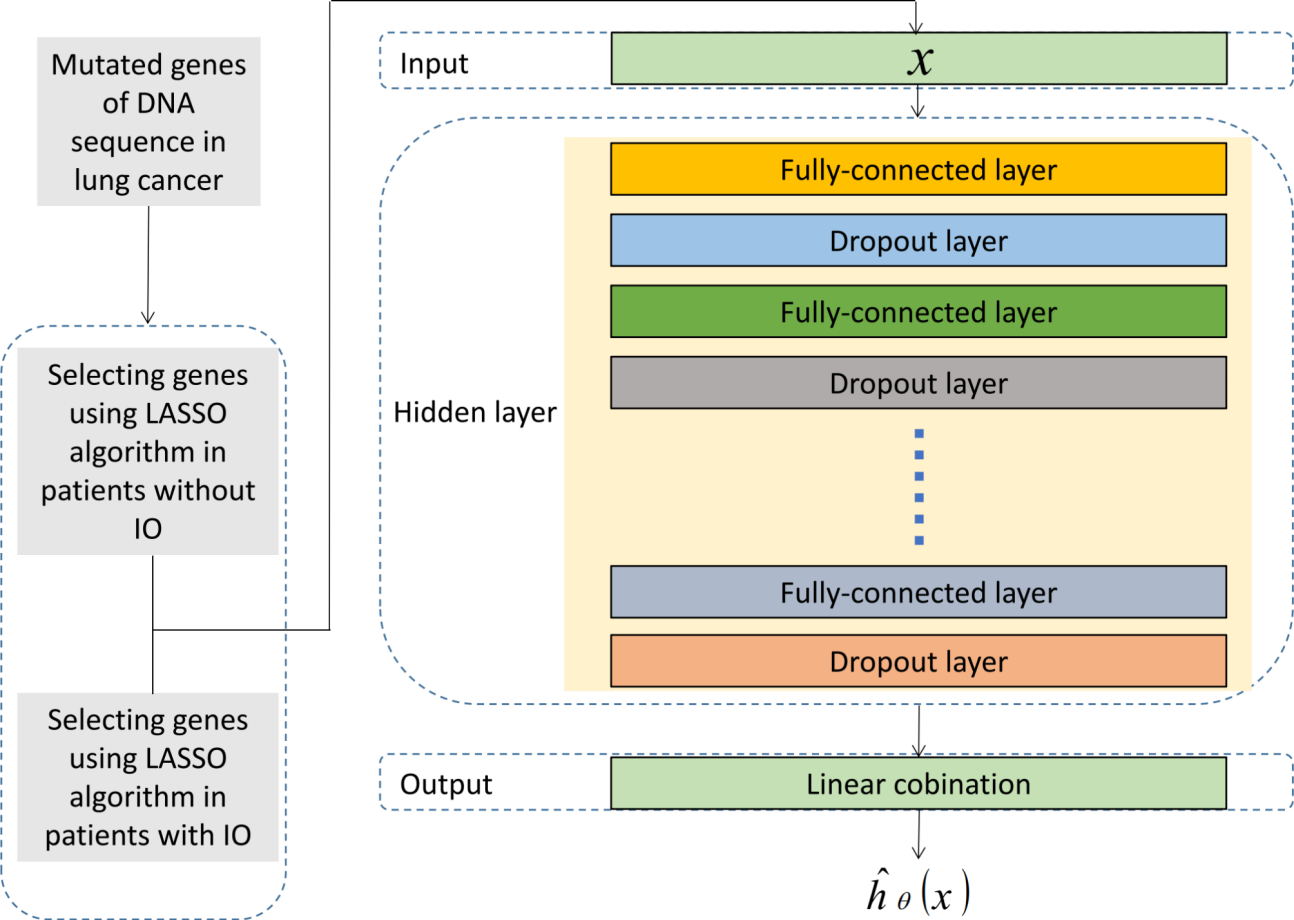
Flowchart of the selection method and the deep neural network architecture. Least absolute shrinkage and selection operator (LASSO) based on five-fold cross-validation was used to select optimal genomics features. The selected genes were imported into the deep learning survival (DLS) model as eigenvectors. The DLS contains multiple hidden layers, weight-decay regularization, rectifying linear units, batch normalization, dropout, and stochastic gradient descent using Nesterov momentum, gradient pruning, and learning rate scheduling. The network output is a single node that estimates the weight of the risk function parameterized through the network. IO, immunotherapy

Based on WES, WGS, and NGS profiling, a TMB ≥ 10 mutations (muts)/Mb or a total number of somatic nonsynonymous mutations ≥ 200 was defined as a high TMB. The tumor cells were considered to have a high PD-L1 expression level when > 50% stained positive. All mutated genes were defined as “1” and wild-type genes were defined as “0.” The optimal mutated genes were selected via LASSO and five-fold cross-validation sampling (Fig. 2). The mutated genes were separately selected to predict OS in patients treated without IO and PFS in patients treated with IO. The selected genes served as input variables for the deep learning model.

### DLS model and implementation

As presented in Fig. 2, the DLS model is a multi-layer perceptron similar to the Faraggi–Simon network (https://github.com/jaredleekatzman/DeepSurv). However, the DLS model comprises multiple additional hidden layers as well as various new methods, including weight-decay regularization, batch normalization, rectifying linear units, dropout, Stochastic gradient descent using gradient pruning, learning rate scheduling, and Nesterov momentum. A single node served as an output of the network that estimated the weight of the risk function parameterized by the network. The loss function was set as a negative log-likelihood function represented by Eq. (1):

1 $$l\left(\theta \right)=-{\sum }_{i,Ei=1}({\widehat{h}}_{\theta }\left(x\right)-{{log}{\sum }_{j\in R\left(Ti\right)}e}^{{\widehat{h}}_{\theta }\left(xj\right)})$$

The selected genes were imported into the DLS model as vectors. The maximum number of epochs was set to 100 to ensure proper implementation of the training procedure. TensorFlow-1.14 in Python (https://www.python.org/) was utilized to implement deep learning. The experiment was conducted in Windows with the following configurations: 3.7 GHz Intel i7-12700KF CPU, NVIDIA GeForce RTX 3090, and 32 GB of RAM.

### Statistical analysis

This study employed the LASSO algorithm, which utilized five-fold cross-validation, to select the optimal non-zero coefficients. A deep learning algorithm-based survival model was applied to predict OS in patients treated without IO and PFS in patients treated with IO. The DLS model’s performance was evaluated in the training and other validation cohorts. The optimal cutoff value for predicting OS or PFS was defined with the X-tile software (https://medicine.yale.edu/lab/rimm/research/Software/). The Kaplan–Meier approach was employed to analyze the PFS and OS curves, which were then plotted with the “survivminer” package. The COX model was based on selected genes using the “rms” package. The accuracies of different models were compared using the C-Index; higher C-indices indicated more accurate model predictive ability. The statistical analyses for this study were performed utilizing R version 3.5.1 (https://www.r-project.org/) and GraphPad Prism 7.01 (https://www.graphpad.com/). Statistical significance was set at *P* < 0.05.

## Results

### Characteristics of individuals treated without and with IO

The basic clinical characteristics of patients with NSCLC treated without IO in the MSK-MET, OncoSG, MSK-CSC, and TCGA-LUAD cohorts are shown in Supplementary Table 1. There were 2064 (54.42%), 150 (50.50%), 461 (49.05%), and 229 (46.93%) male patients in the MSK-MET, OncoSG, MSK-CSC, and TCGA-LUAD cohorts, respectively. In the MSK-MET, OncoSG, and TCGA-LUAD cohorts, 2060 (54.31%), 183 (61.62%), and 325 (66.60%) patients were aged > 60 years. Most patients (62.29%) were never smokers in the OncoSG cohort. Moreover, 817 (21.54%), 24 (8.08%), 218 (23.20%), and 173 (35.45%) patients, respectively, had a high TMB (≥ 200 or > 20 muts/Mb) and the TMB status was diverse in the different populations.

The basic clinical features of individuals with NSCLC treated with IO in the MIND, MSKCC, and POPLAR/OAK cohorts are presented in Supplementary Tables 2, with 112 (45.53%), 172 (49.28%), and 275 (78.80%) male patients, respectively. In the 3 cohorts, 190 (77.23%), 222 (67.15%), and 265 (75.93%) patients, respectively, were aged > 60 years. Most individuals in the MSKCC (80.51%) and POPLAR/OAK (80.51%) cohorts were current or ever smokers. Additionally, in the 3 cohorts, 15 (3.50%), 71 (20.34%), and 175 (27.22%) patients, respectively, had a high TMB (≥ 200 or > 20 muts/Mb) with diverse TMB status among the populations. In the MIND, MSKCC, and POPLAR/OAK cohorts, 119 (48.37%), 43 (12.32%), and 59 (12.33%) individuals, respectively, had positive PD-L1 expression (> 1%). In these 3 cohorts, 81 (32.93%), 218 (62.46%), and 295 (68.76%) patients, respectively, achieved durable clinical benefits.

### Selection of mutational genes associated with prognosis in patients with and without IO

**Fig. 3:**
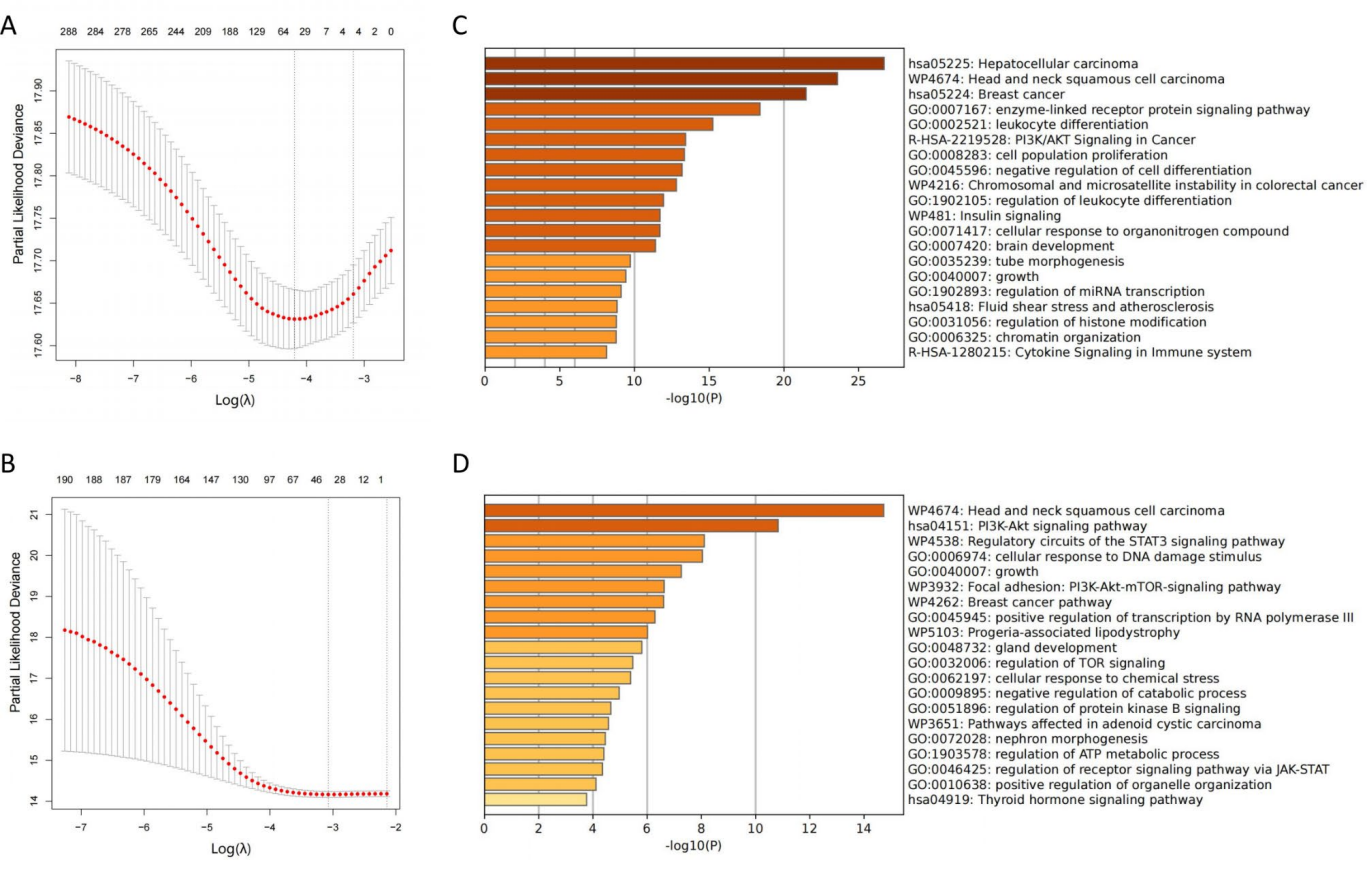
Least absolute shrinkage and selection operator (LASSO) selection of genes and pathway analysis. **(a, b)** Optimal somatic mutations selected in patients with non-small cell lung cancer who did or did not receive immunotherapy. **(c, d)** Enrichment analysis of somatic mutations and different signaling pathways

Based on the five-fold cross-validation, LASSO was applied to select the optimal mutational genomics from the MSK-MET cohort (training). In total, 45 somatic mutations were selected (Fig. 3a; Supplementary Table 3). High-mutational-frequency genes, such as *TP53*, *EGFR*, *STK11*, *KRAS*, and *KEAP1*, were selected in the MSK-MET cohort (training). Similarly, in the MIND cohort, 27 somatic mutations were identified in patients with lung cancer treated with IO (Fig. 3b). The Kyoto Encyclopedia of Genes and Genomes analysis revealed that the 45 mutational genes were associated with various cancer pathways, including hepatocellular carcinoma, head and neck squamous cell carcinoma, and breast cancer (false discovery rate [FDR]: *P* < 0.001; Fig. 3c). An association was observed between the 27 mutational genes for predicting PFS in the MIND cohort and immunology signaling pathways (FDR: *P* < 0.001; Fig. 3d), including the regulatory circuits of the STAT3 signaling pathway and cellular response to DNA damage stimuli. Subsequently, a panel of 45 mutational genes was employed to train the model in predicting OS in the MSK-MET cohort (training) treated without IO based on deep learning algorithms. The model was the retrained using a panel of 27 mutational genes to predict PFS in the MIND cohort treated with IO.

### Training and testing the DLS model for OS in patients treated without IO

**Fig. 4:**
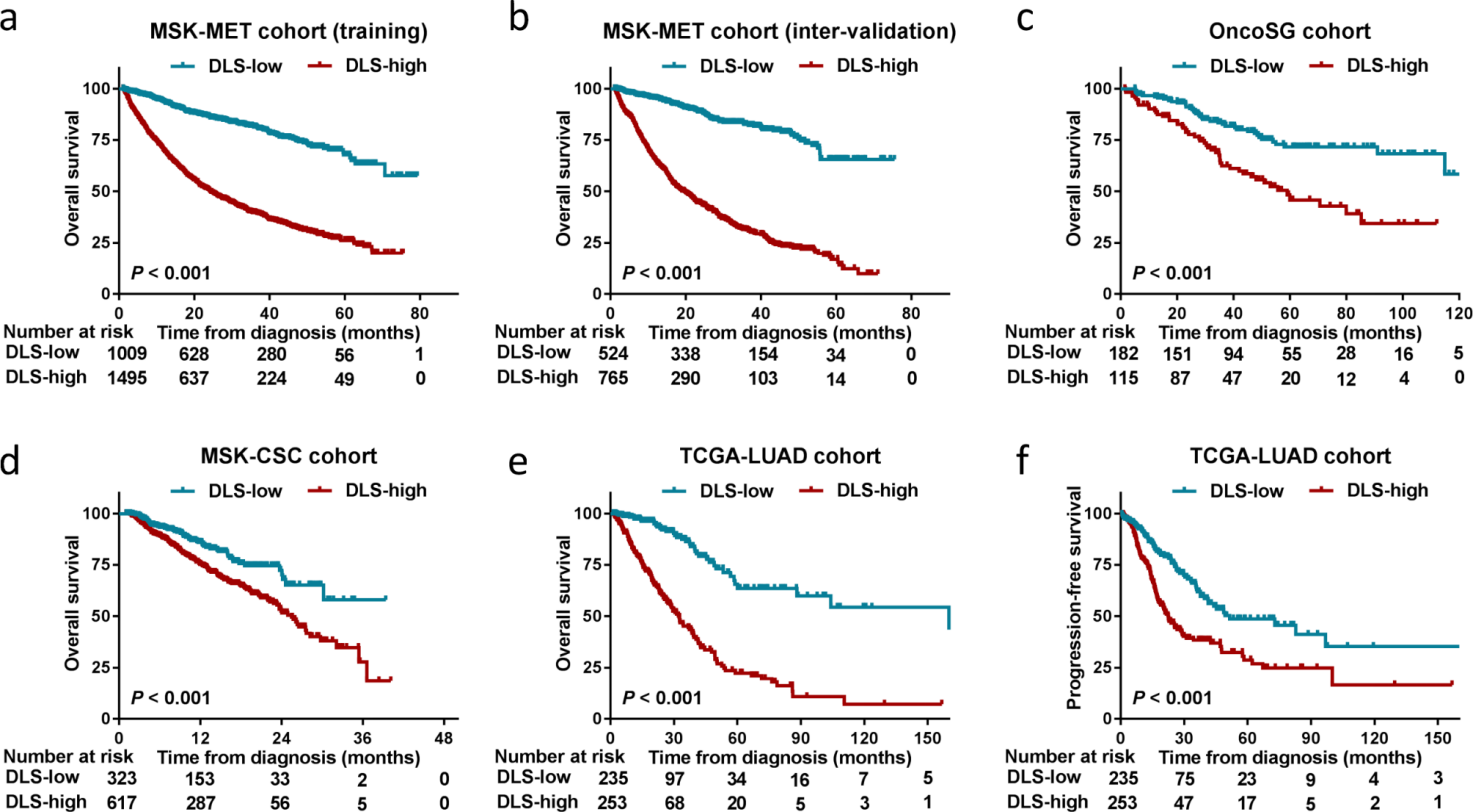
Development and validation of the deep learning survival (DLS) model for overall survival (OS). **(a, b)** DLS model comprising 45 selected genes in the training MSK-MET cohort was tested in the inter-validation MSK-MET cohort. **(c, d)** OS curves of patients from the OncoSG and MSK-CSC cohorts. **(e, f)** OS and progression-free survival curves of patients from the TCGA-LUAD cohort

The DLS model was run using the TensorFlow 1.14 platform (https://tensorflow.google.cn/install/source). The MSK-MET cohort (training) was trained in 100 epoch processes, and the MSK-MET cohort (inter-validation) was used for validation (Supplementary Fig. 1). The OncoSG, MSK-CSC, and TCGA-LUAD cohorts were tested using the trained DLS model. According to the cutoff value (0.50) of DLS scores as the X-tile (https://en.freedownloadmanager.org/Windows-PC/X-tile-FREE.html), individuals with NSCLC treated without IO were stratified into high (> 0.50) and low (≤ 0.50) DLS groups. The high DLS group had a shorter median OS than the low DLS group (24.18 months vs. not reached [NR]; hazard ratio [HR] = 4.13 [3.66–4.67], *P* < 0.001; Fig. 4a) in the MSK-MET cohort (training) treated without IO (Fig. 4a). In the MSK-MET cohort (inter-validation), the high DLS group also had a shorter median OS than the low DLS group (19.68 months vs. NR; HR = 5.71 [4.85–6.72], *P* < 0.001; Fig. 4b). In the OncoSG and MSK-CSC cohorts, the high DLS group was validated and had a shorter median OS than the low DLS group (OncoSG: 59.00 months vs. NR; HR = 2.37 [1.54–3.67], *P* < 0.001; MSK-CSC: 25.40 months vs. NR; HR = 1.82 [1.40–2.35], *P* < 0.001, Fig. 4c, d). Likewise, in the TCGA-LUAD cohort, the high DLS group had a shorter median OS and PFS than the low DLS group (OS: 32.45 vs. 63.10 months; HR = 4.63 [3.43–6.25], *P* < 0.001; PFS: 22.49 vs. 51.55 months; HR = 2.08 [1.58–2.75], *P* < 0.001, Fig. 4e, f).

### DLS model fine-tuning and retraining for PFS in patients treated with IO

**Fig. 5:**
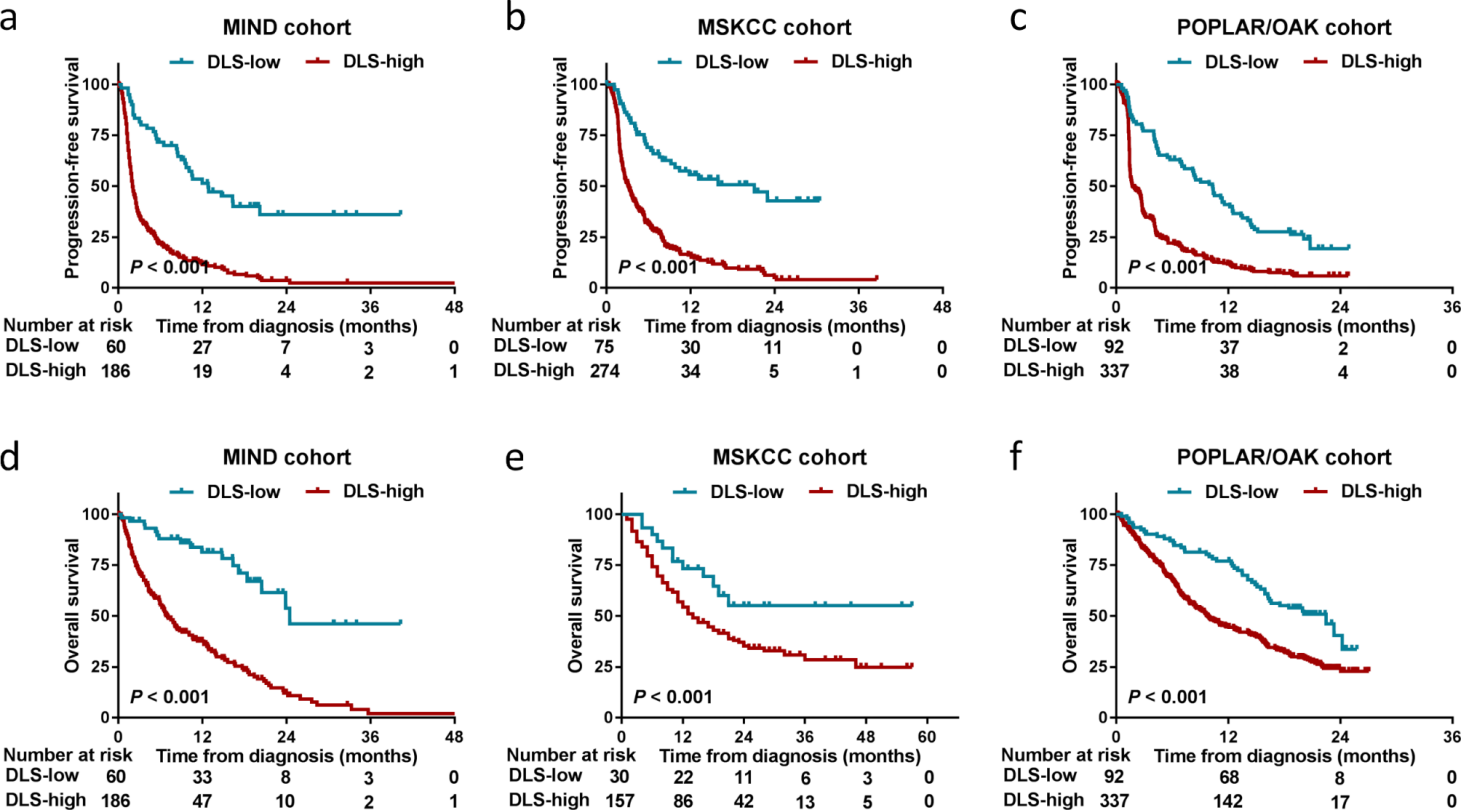
Development and validation of the deep learning survival (DLS) model for progression-free survival (PFS). **(a–c)** DLS model for predicting PFS comprising 27 selected genes was constructed in the MIND cohort and validated in the MSKCC and POPLAR/OAK cohorts. **(d–f)** Overall survival curves of patients in the MIND, MSKCC, and POPLAR/OAK cohorts

In determining the prognosis of patients receiving anti-PD-1 therapy, the DLS model was fine-tuned and retrained via 27 selected mutational genes. Individuals with NSCLC treated with IO were categorized into the high (> 0.50) and the low (≤ 0.50) DLS groups. The low DLS group had a longer median PFS than the high DLS group (12.80 vs. 2.00 months; HR = 3.41 [2.58–4.98], *P* < 0.001; Fig. 5a) in the MIND cohort treated with IO. In the MSKCC and POPLAR/OAK cohorts, the low DLS group exhibited better PFS than the high DLS group (both *P* < 0.001; Fig. 5b, c). The DLS model’s ability to predict OS in the MIND cohort was validated; the low DLS group had a considerably longer median OS duration (24.50 vs. 7.00 months; HR = 4.34 [3.11–6.06], *P* < 0.001) than that of the high DLS group (Fig. 5d). The low DLS group had better OS than that of the high DLS group in the MSKCC and POPLAR/OAK cohorts (both *P* < 0.001; Fig. 5e, f).

### Comparison of the DLS model with clinical features and the COX model

**Fig. 6:**
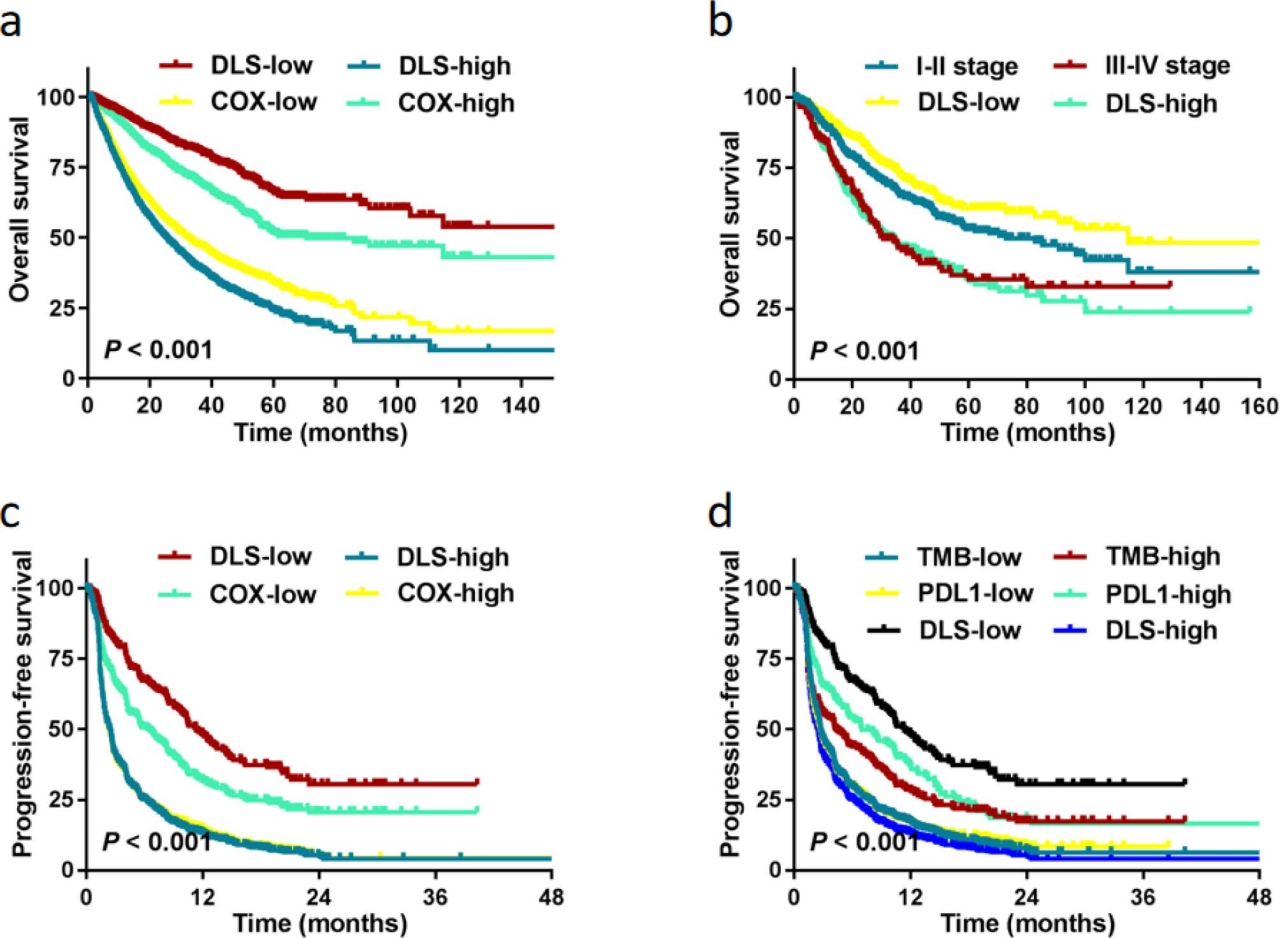
Deep learning survival (DLS) compared with the COX model and other clinical predictive methods. **(a, b)** Comparison of the DLS model with the COX model and clinical staging in all cohorts treated without IO in predicting OS. **(c, d)** Comparison of the DLS model with the COX model, tumor mutational burden (TMB), and programmed death-ligand 1 (PD-L1) expression in all cohorts with IO in predicting PFS.

In all 4 cohorts treated without IO, a routine model was developed using the COX method based on the selected panel of 45 mutational genes. The high COX group had a longer median OS than that of the low COX group (70.67 vs. 32.00 months; HR = 0.48 [0.44–0.53], *P* < 0.001; Fig. 6a). The C-index of the DLS model was significantly higher than that of the TNM stage or COX model (0.74 vs. 0.60 vs. 0.63). The low DLS group had a better OS than that of the TNM stage I–II groups (*P* < 0.010; Fig. 6b). In all three cohorts (MIND, MSKCC, and POPLAR/OAK) treated with IO, the low COX group had a longer median PFS than the high COX group (6.34 vs. 2.37 months; HR = 0.53 [0.47–0.61], *P* < 0.001; Fig. 6c). The C-index of the DLS model was significantly higher than that of the COX model (0.70 vs. 0.61). The low DLS group had a better PFS than that of the high PD-L1 group (*P* < 0.001; Fig. 6d) and high TMB group (*P* < 0.001; Fig. 6d). The C-index of the DLS model was significantly higher than that of the PD-L1 and TMB groups (0.70 vs. 0.55 vs. 0.54).

## Discussion

In this study, deep learning methods were employed using multi-center sequencing data to develop predictive models for OS or PFS in individuals with NSCLC from several cohorts treated with or without IO. As per our knowledge, this is the largest study to determine prognosis based on sequencing data from patients with NSCLC. Moreover, to prevent over-fitting of the DLS model, the LASSO algorithm was initially utilized to select optimal genes. Ultimately, 45 somatic mutations were selected to predict OS in patients treated without IO. The DLS model was validated in the MSK-MET (inter-validation), OncoSG, MSK-CSC, and TCGA-LUAD cohorts. After fine-tuning and retraining the parameters, a DLS model based on 27 somatic mutations was applied to predict PFS in the MIND cohort treated with IO. The DLS model was also validated in the MSKCC and POPLAR/OAK cohorts. Further, the COX model and TNM staging were compared with the DLS model in all cohorts treated without IO, revealing that the DLS model had the highest C-index. The DLS model also exhibited superior predictive performance compared to the TMB, PD-L1 expression, and COX models in all cohorts.

Although the WGS, NGS, and WES databases have been used increasingly and extensively in cancer research, most studies have focused on several gene panels or sole driver mutational genes. Consequently, the large amount of sequencing data available is not being efficiently utilized, particularly for somatic mutations [23–30]. In contrast, the current study focused primarily on employing a relatively small panel of mutational genes to develop a robust predictive model for disease prognosis. To the best of our knowledge, this is the first study to use deep learning to train somatic mutations for predicting OS in patients treated without IO or routine images. Importantly, different sequencing methods did not affect predictive ability. However, additional research is needed to investigate whether DLS can classify OS prediction utilizing a large amount of data obtained from WES, NGS, or WGS without relying on simple somatic mutations. The genomic sequencing data analyzed in this study were obtained from tumor DNA. Moreover, the training model was validated with data from the other four cohorts (MSK-MET, OncoSG, MSK-CSC, and TCGA-LUAD), all of which underwent tumor tissue sequencing. Based on these results, it can be concluded that the DLS model is a feasible and robust method for accurately predicting the OS of patients with NSCLC. Moreover, the DLS model could predict PFS in the TCGA-LUAD cohort undergoing surgery, indicating that this model can be applied to predict recurrence time via sequencing data.

Several machine-learning models have been used to predict PFS and OS in patients who received IO [31–33]. However, herein, a deep learning algorithm based on somatic mutations was used for the first time to directly predict PFS. In this study, patients with low DLS had significantly better PFS and OS than did those with high DLS in the MIND, MSKCC, and POPLAR/OAK cohorts. These findings imply that the DLS model could efficiently evaluate clinical prognosis in patients with NSCLC treated with or without IO. In contrast, TMB and PD-L1 expression exhibited unsatisfactory outcomes in predicting PFS and OS in the three cohorts. It is hypothesized that using various detection platforms or different cutoff values for TMB might have led to an uncertain predictive impact. Indeed, the PD-L1 assay may have employed diverse reagents from several manufacturers [34, 35], and the expression levels of PD-L1 from different tumor regions may have differed [36]. Hence, the DLS model is a viable tool that can overcome the drawbacks of TMB or PD-L1 expression levels to predict clinical outcomes in patients with NSCLC treated with IO.

Employing deep learning to predict disease prognosis, involving medical images or clinical features, has gradually been introduced in cancer research [37–39]. However, acquiring a large database of clinical features to train models is difficult, especially regarding genomic mutations and patients with cancer who receive IO. Transfer learning is a promising strategy for addressing the issue of small sample sizes [40]. The current study used transfer learning to train the DLS model with similar predictive objectives. The DLS model for predicting OS in patients treated without IO was first trained using larger sequencing data after selecting optimal somatic mutations, avoiding overfitting during training. Although the deep learning method had more parameters and complexities, it also had a higher and more consistent ability to predict OS than the COX model (C-index: 0.74 vs. 0.63). Moreover, deep learning based on genomic mutations could better reflect the prognostic status than simple clinical staging. This indicates that analysis of sequencing mutation information would greatly improve the development of molecular typing in lung cancer. Nevertheless, large-scale sequencing data is difficult to acquire, particularly for patients receiving IO or chemotherapy plus IO. In our study, after the DLS model was trained in patients who did not receive IO, it was retrained using a smaller dataset (MIND cohort), indicative of transfer learning. This method could allow for training with smaller-scale mutational data in other cancers while maintaining model stability. The DLS model also presented higher predictive ability than that of the COX model in patients who received IO (C-index: 0.70 vs. 0.61). Therefore, this novel deep-learning algorithm has the capacity to increase the identified associations between prognosis and gene status greatly.

This study has few limitations. First, although the study included many patients from numerous centers, several clinical variables (e.g., PFS and tumor biomarkers) were missing in the MSK-MET, OncoSG, and MSK-CSC cohorts. Therefore, the DLS model could not incorporate these clinical variables to optimize predictive performance further. Additionally, although a panel of selected somatic mutations based on WES, WGS, or NGS data was employed, copy number variation, mRNA expression, radiomics, and pathology grade were not utilized to predict OS and PFS. A deep learning method based on a multi-omics model could be evaluated. Furthermore, circulating tumor DNA analysis of peripheral blood samples is a noninvasive approach only conducted in the POPLAR/OAK cohort. Hence, the predictive performance of the DLS model for prognosis based on circulating tumor DNA could be further investigated.

## Conclusions

Herein, deep learning based on a panel of mutational genes served as a novel and reliable algorithm for determining the prognosis in patients with NSCLC who did or did not receive IO. The DLS model can predict OS and PFS better than the COX model, TNM staging, TMB, or PD-L1 expression. Our findings provide new insights for predicting clinical outcomes in patients with NSCLC based on the WGS, NGS, and WES databases. This new deep learning algorithm from high-throughput sequencing can be exploited to inform pan-cancer clinical decisions.

### Electronic supplementary material

Below is the link to the electronic supplementary material.

Supplementary Material 1: **Supplementary Table 1.** Characteristics of the patients who did not receive immunotherapy. **Supplementary Table 2.** Characteristics of the patients who received immunotherapy. **Supplementary Table 3.** Selected mutational genes associated with prognosis in patients who did or did not receive immunotherapy. **Supplementary Fig. 1.** Training process for the deep learning survival model based on 45 somatic mutations for predicting overall survival in the MSK-MET cohort (training). KM, Kaplan–Meier. **Supplementary Fig. 2.** Training process for the deep learning survival model based on 27 somatic mutations for predicting progression-free survival in the MIND cohort. KM, Kaplan–Meier.

## Data Availability

The data supporting the findings of this study are available upon request from the corresponding author.

## Abbreviations

HR: Hazard ratio
IO: Immunotherapy
MSKCC: Memorial Sloan Kettering Cancer Center
NGS: Next-generation sequencing
NSCLC: Non-small cell lung cancer
NR: Not reached
OS: Overall survival
PD-L1: Programmed death-ligand 1
PFS: Progression-free survival
TMB: Tumor mutational burden
TNM: Tumor-node-metastasis
WES: Whole-exome sequencing
WGS: Whole-genome sequencing
