# Supplementary material for: Determining the prognosis of Lung cancer from mutated genes using a deep learning survival model: a large multi-center study
Source: Cancer Cell Int. 2023 Nov 4;23:262. doi: 10.1186/s12935-023-03118-y (PMC10625246; doi:10.1186/s12935-023-03118-y)
Supplement: Supplementary file 1 — Supplementary Material 1: Supplementary Table 1. Characteristics of the patients who did not receive immunotherapy. Supplementary Table 2. Characteristics of the patients who received immunotherapy. Supplementary Table 3. Selected mutational genes associated with prognosis in patients who did or did not receive immunotherapy. Supplementary Fig. 1. Training process for the deep learning survival model based on 45 somatic mutations for predicting overall survival in the MSK-MET cohort (training). KM, Kaplan–Meier. Supplementary Fig. 2. Training process for the deep learning survival model based on 27 somatic mutations for predicting progression-free survival in the MIND cohort. KM, Kaplan–Meier. [file 12935_2023_3118_MOESM1_ESM.docx]

Supplementary Material

Determining the prognosis of lung cancer from mutated genes using a deep learning survival model: A large multi-center study

Jie Peng^1^*^†^, Lushan Xiao^2†^, Hongbo Zhu^3†^, Lijie Han^4^, and Honglian Ma^5^

^1^Department of Medical Oncology, The Second Affiliated Hospital, Guizhou Medical University, Kaili, China

^2^Hepatology Unit and Department of Infectious Diseases, Nanfang Hospital, Southern Medical University, Guangzhou, China

^3^Department of Medical Oncology, The First Affiliated Hospital, Hengyang Medical School, University of South China, Hengyang, China

^4^Department of Hematology, The First Affiliated Hospital of Zhengzhou University, Zhengzhou, China

^5^Department of Radiation Oncology, Cancer Hospital of the University of Chinese Academy of Sciences, Hangzhou, China

^†^These authors contributed equally to this work.

# * Correspondence: Jie Peng: [sank44@sina.com](mailto:sank44@sina.com)

# Supplementary Figures and Tables

## Supplementary Figures


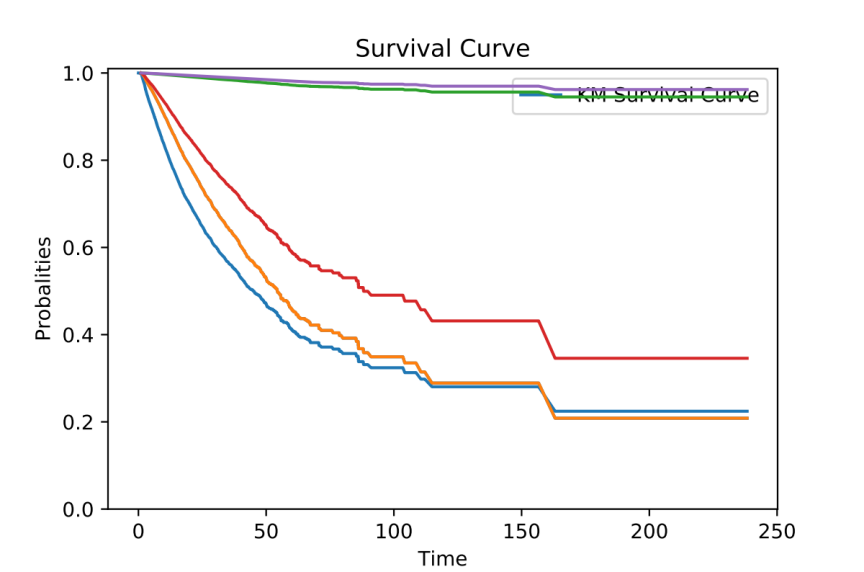


**Supplementary Figure 1.** Training process for the deep learning survival model based on 45 somatic mutations for predicting overall survival in the MSK-MET cohort (training). KM, Kaplan–Meier.


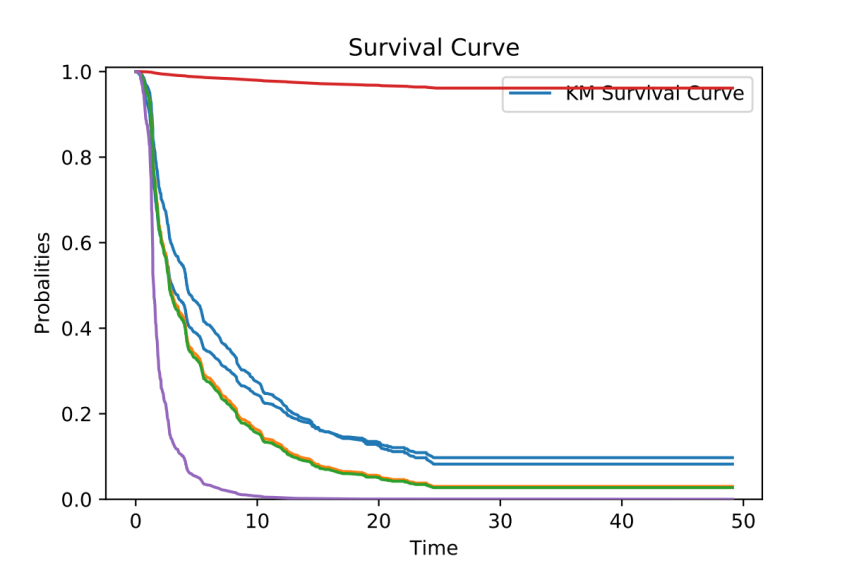


**Supplementary Figure 2.** Training process for the deep learning survival model based on 27 somatic mutations for predicting progression-free survival in the MIND cohort. KM, Kaplan–Meier.

## Supplementary Tables

**Supplementary Table 1. Characteristics of the patients who did not receive immunotherapy**

| **Variable** | **MSK-MET cohort**  **(*n* = 3793)** | **OncoSG**  **cohort**  **(*n* = 297)** | **MSK-CSC cohort**  **(*n* = 940)** | **TCGA-LUAD cohort**  **(*n* = 488)** |
| --- | --- | --- | --- | --- |
| Sex |  |  |  |  |
| Female | 2339 (45.58%) | 147 (49.50%) | 479 (50.95%) | 259 (53.07%) |
| Male | 2064 (54.42%) | 150 (50.50%) | 461 (49.05%) | 229 (46.93%) |
| Age (years) |  |  |  |  |
| ≤ 60 | 879 (23.17%) | 114 (38.38%) | - | 153 (31.35%) |
| > 60 | 2060 (54.31%) | 183 (61.62%) | - | 325 (66.60%) |
| NA | 854 (22.52%) | - | - | 10 (2.05%) |
| Smoker |  |  |  |  |
| Yes | - | 108 (36.36%) | - | - |
| No | - | 185 (62.29%) | - | - |
| NA | - | 4 (1.35%) | - | - |
| TMB |  |  |  |  |
| Low | 2976 (78.46%) | 273 (91.92%) | 722 (76.80%) | 315 (64.55%) |
| High | 817 (21.54%) | 24 (8.08%) | 218 (23.20%) | 173 (35.45%) |

Data are presented as n (%).

NA, not available; TMB, tumor mutational burden.

**Supplementary Table 2. Characteristics of the patients who received immunotherapy**

| **Variable** | **MIND cohort**  **(*n* = 246)** | **MSKCC cohort**  **(*n* = 349)** | **POPLAR/OAK cohort**  **(*n* = 429)** |
| --- | --- | --- | --- |
| Sex |  |  |  |
| Female | 134 (54.47%) | 177 (50.72%) | 154 (21.20%) |
| Male | 112 (45.53%) | 172 (49.28%) | 275 (78.80%) |
| Age (years) |  |  |  |
| ≤ 60 | 56 (22.77%) | 127 (36.39%) | 164 (24.07%) |
| > 60 | 190 (77.23%) | 222 (67.15%) | 265 (75.93%) |
| Smoker |  |  |  |
| Yes | - | 281 (80.51%) | 352 (80.51%) |
| No | - | 68 (19.49%) | 77 (19.49%) |
| TMB |  |  |  |
| Low | 414 (96.50%) | 278 (79.66%) | 254 (72.78%) |
| High | 15 (3.50%) | 71 (20.34%) | 175 (27.22%) |
| PD-L1 |  |  |  |
| Negative | 127 (51.63%) | 152 (43.55%) | 263 (75.36%) |
| Positive | 119 (48.37%) | 43 (12.32%) | 59 (12.33%) |
| NA | - | 154 (44.13%) | 107 (12.31%) |
| DCB |  |  |  |
| Yes | 81 (32.93%) | 218 (62.46%) | 295 (68.76%) |
| No | 165 (67.07%) | 131 (37.54%) | 134 (31.24%) |

Data are presented as n (%).

DCB, durable clinical benefit; NA, not available; PD-L1, programmed death-ligand 1; TMB, tumor mutational burden.

**Supplementary Table 3. Selected mutational genes associated with prognosis in patients who did or did not receive immunotherapy**

| **Group** | **Selected mutational genes** |
| --- | --- |
| **MSK-MET cohort (training)** | *AKT3, ARID1A, BAP1, BCOR, BMPR1A, CBL, CSF1R, CTNNB1, DDR2, DNMT3A, EGFR, EPHA3, EPHA7, ERBB2, ERBB3, ESR1, GATA2, GSK3B, INPP4A, IRS1, JAK3, KEAP1, KRAS, MAP2K1, MAP3K1, MED12, MEN1, MLH1, MTOR, NFE2L2, NRAS, PAX5, PGR, PIK3C2G, RB1, ROS1, SMARCA4, STK11, TGFBR2, TP53, U2AF1, PIK3R3, RUNX1, SF3B1, SMARCB1* |
| **MIND cohort** | *ATM, ATRX, CUL3, DOT1L, EGFR, EPHA3, ERBB4, FLT4, FUBP1, INHBA, INPP4B, KEAP1, MCL1, MED12, NCOR1, NRAS, NTRK2, PARP1, PDGFRB, POLD1, POLE, PTPRD, RICTOR, RPTOR, SF3B1, SMAD4, STK11* |
